# Plant-plant competition outcomes are modulated by plant effects on the soil bacterial community

**DOI:** 10.1038/s41598-017-18103-5

**Published:** 2017-12-19

**Authors:** S. Hortal, Y. M. Lozano, F. Bastida, C. Armas, J. L. Moreno, C. Garcia, F. I. Pugnaire

**Affiliations:** 1 Estación Experimental de Zonas Áridas, Consejo Superior de Investigaciones Científicas (EEZA-CSIC), Carretera de Sacramento s/n, E-04120 La Cañada de San Urbano, Almería Spain; 2 Hawkesbury Institute for the Environment, Western Sydney University, Locked Bag 1797, Penrith, NSW 2751 Australia; 3 Centro de Edafología y Biología Aplicada del Segura, Consejo Superior de Investigaciones Científicas (CEBAS-CSIC), Campus Universitario de Espinardo, P.O. Box 164, E-30100 Murcia, Spain; 4 Freie Universität Berlin, Institut für Biologie, Plant Ecology, D-14195 Berlin, Germany; 5 Berlin-Brandenburg Institute of Advanced Biodiversity Research (BBIB), D-14195 Berlin, Germany

## Abstract

Competition is a key process that determines plant community structure and dynamics, often mediated by nutrients and water availability. However, the role of soil microorganisms on plant competition, and the links between above- and belowground processes, are not well understood. Here we show that the effects of interspecific plant competition on plant performance are mediated by feedbacks between plants and soil bacterial communities. Each plant species selects a singular community of soil microorganisms in its rhizosphere with a specific species composition, abundance and activity. When two plant species interact, the resulting soil bacterial community matches that of the most competitive plant species, suggesting strong competitive interactions between soil bacterial communities as well. We propose a novel mechanism by which changes in belowground bacterial communities promoted by the most competitive plant species influence plant performance and competition outcome. These findings emphasise the strong links between plant and soil communities, paving the way to a better understanding of plant community dynamics and the effects of soil bacterial communities on ecosystem functioning and services.

## Introduction

Plant-plant competition plays a key role in defining community structure and dynamics^1^. The outcomes of this process are often mediated by the availability of nutrients and water in the soil^2^. However, the role of soil microorganisms on plant-plant competition is less understood^3,4^ despite evidence for strong links between above- and belowground biota^5,6^. The interaction between plants and associated soil microbial communities is so close that they can be considered a whole entity^7^ that jointly responds to environmental conditions^8^ and which is subject to selection^9^. Plants shape their rhizosphere microbial communities through changes in soil temperature, moisture, physical structure, litter quality, and root exudates^2,10–13^. Soil microbial communities, in turn, influence plant community structure by altering plant performance and functional traits^14–16^, which affect ecosystem functioning through their effects on nutrient cycles and productivity^11,17^.

Because of this close interaction, assessing the role of soil communities on plant-plant interactions should provide a more realistic view of plant community dynamics and its consequences for ecosystem functioning. In this regard, it has been shown that soil bacteria influence, for instance, facilitation^13,14^, a key process in community assembly. However, there is less evidence on whether they can mediate the outcome of plant competition^3,4,18^. We formulated the hypotheses that 1) under intraspecific competition, each plant species will develop a specific bacterial community in its rhizosphere with different composition and activity as a result of different root traits that foster contrasting communities; 2) interspecific competition between two co-existing plant species, similar in size and habitat preferences, will have measurable effects on plant survival, growth and functional traits; and 3) when the two species interact, the final microbial community will resemble the community of the most competitive plant species.

To test these hypotheses, we monitored survival and performance of individuals of two plant species growing with an individual of the same (intraspecific competition) or the other species (interspecific competition) in a greenhouse experiment, using a common soil. We characterized the composition of soil bacterial communities in the different treatments at the end of the experiment by using 16S rDNA sequencing. We also measured enzyme activities to determine both the total microbial activity (through dehydrogenase activity) and the performance of the biogeochemical processes related with carbon (C), nitrogen (N) and phosphorus (P) (β-glucosidase, urease and alkaline phosphatase activities, respectively). Our target species were *Maytenus senegalensis* subsp. *europea* Rivas and *Lycium intricatum* Boiss (*Maytenus* and *Lycium* hereafter), two shrub species coexisting in semiarid environments in southern Spain. The two species have differential effects on their associated plant communities^2,19^, suggesting contrasted effects on microbial communities. Thus, while *Maytenus* is a facilitator shrub that creates favourable conditions for an understorey community of beneficiary species^19^, *Lycium* induces a large decrease in photosynthetically active radiation and soil moisture in its understorey^2^. Because competition is a common process happening whenever two individuals co-occur^1,20,21^, the results presented here are applicable to other pairs of co-existing plant species and contribute to better understanding plant community assembly.

## Results and Discussion

### Plant survival and growth

**Figure 1:**
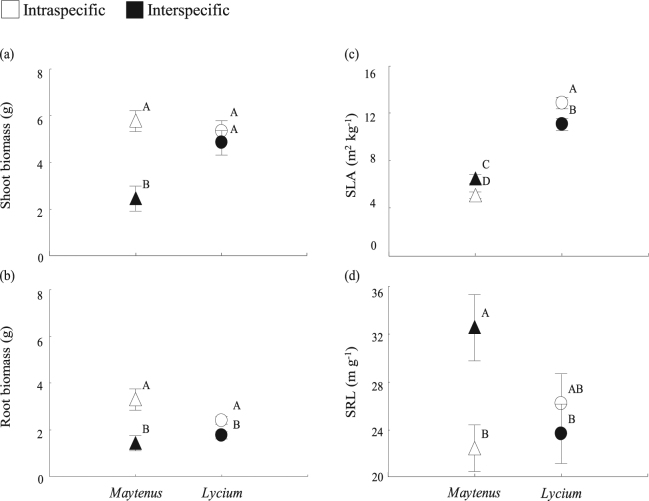
*Maytenus* plants grew less and showed increased SLA and SRL when growing with *Lycium*. Shoot (**a**) and root (**b**) mass, specific leaf area (SLA; **c**) and specific root length (SRL; **d**) in *Maytenus* and *Lycium* plants growing under intra- or interspecific interaction. Different letters in a graph indicate significant differences (p < 0.05) among treatments. Data are mean ± 1 SE; n = 6.

Interspecific competition altered plant survival and growth, supporting our second hypothesis. This was true in particular for *Maytenus* individuals. *Maytenus* mortality started 8 months after the beginning of the experiment. By month 12, *Maytenus* mortality was 60% higher under interspecific competition than under intraspecific competition (Supplementary Fig. S1 and Supplementary Table S1). By contrast, all *Lycium* individuals survived regardless of treatment. At the end of the experiment, *Maytenus* individuals also had lower aboveground mass under interspecific than under intraspecific competition (Fig. 1a, Supplementary Table S2). Both species had less root mass in the interspecific treatment than in the intraspecific treatment (Fig. 1b), suggesting strong belowground competition.

### Plant traits

**Figure 2:**
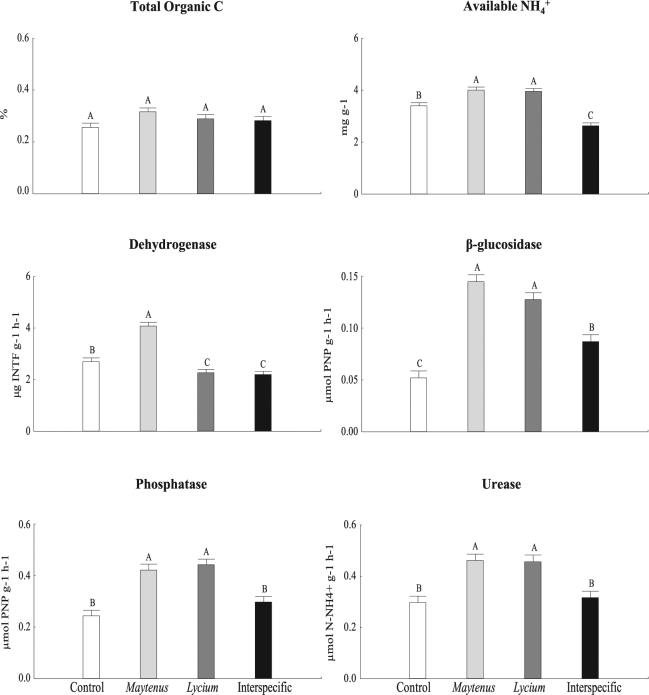
Soil enzyme activity was lower in interspecific soils than in intraspecific ones. Soil TOC, available NH_4_^+^ and enzyme activities in soils of the different plant interaction treatments, *i.e*. without plants (control), soils with two *Maytenus* individuals (*Maytenus*-intraspecific), with two *Lycium* individuals (*Lycium*-intraspecific) or with one individual of each plant species (interspecific). Different letters in a graph indicate significant differences (p < 0.05) among treatments. Data are mean ± 1 SE; n = 6. Graph columns are coloured according to the four different plant interaction treatments (control, *Maytenus, Lycium*, interspecific).

As expected, we found changes in key plant functional traits as a response of one species interacting with another (Fig. 1, Supplementary Table S2). This supports the hypothesis that plant interactions are major drivers of trait plasticity^22^. The specific leaf area (SLA) has been shown to respond to the presence of a competitor^23^, and in our study, both *Maytenus* and *Lycium* individuals changed SLA when in interspecific competition in contrast to intraspecific competition (Fig. 1c). Interestingly, these changes in SLA followed opposite directions for the two plant species. On one hand, *Lycium* responded with a decrease in SLA under interspecific competition compared to intraspecific competition, which is often related to increased leaf longevity and C investment in secondary compounds^24^. On the other hand, *Maytenus* responded with an increase in SLA under interspecific competition, which is often linked to an increase in leaf N concentration and photosynthetic rate^24^. *Maytenus* individuals also increased specific root length (SRL), i.e. produced thinner roots, when growing with *Lycium* (Fig. 1d). The increase in both SLA and SRL in *Maytenus* suggests a strategy to maximize N uptake^24^, a nutrient that was most limited in the interspecific interaction treatment (available NH_4_^+^; Fig. 2). Thinner root production is often triggered by the presence in the soil of root exudates of other species^25^. Our results support the tenet that plants display different functional responses to optimize performance during competition^26^. Because trait changes imply changes in ecosystem properties^11^, the consequences of a competitive environment might translate into altered ecosystem functioning through species turnover.

### Soil enzyme activities

There was a decrease in most soil enzyme activity in the interspecific treatment compared to soils in the intraspecific treatment, either with only *Lycium* or only *Maytenus* (Fig. 2, Supplementary Tables S2 and S3). This lower activity should be an indirect consequence of lower plant growth and less production of root exudates that stimulate bacterial activity. Indeed, responses to plant competitors are likely to alter the quantity, quality and availability of resources supplied by a host plant to its microbiome^27^. The reduced soil N in pots of the interspecific interaction compared to control pots with no plants (while having similar levels of urease activity, Fig. 2) suggests that plants under interspecific competition actively took up most of the available N. Consequently, it is very likely that soil microbes were also competing amongst themselves and also with the plants for N^28^. The decrease in microbial activity, and therefore the slow nutrient cycling in the interspecific treatment, may have contributed to the observed poor *Maytenus* performance in this treatment. Our results support the suggestion that root exudates, nutrient availability and soil microbial community form a tripartite relationship that simultaneously affects plant growth and competitive ability^26^.

### Diversity and composition of bacterial communities

**Figure 3:**
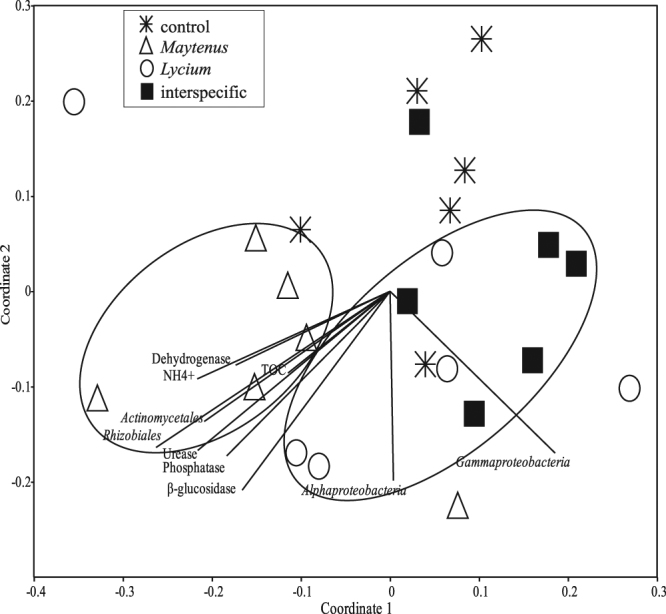
The soil bacterial community in the interspecific treatment was similar to *Lycium* but different to *Maytenus* soils. Ordination of soil bacterial community composition by Non-Metric Multidimensional Scaling (NMDS) in the different plant interaction treatments, *i.e*. soils without plants (control), with two *Maytenus* individuals (*Maytenus*-intraspecific), with two *Lycium* individuals (*Lycium*-intraspecific) or with one individual of each plant species (interspecific). Samples are coded by plant interaction treatment, in particular: asterisks = control, triangles = *Maytenus*-intraspecific, circles = *Lycium*-intraspecific; black filled squares = interspecific; n = 6. Stress = 0.2.

**Figure 4:**
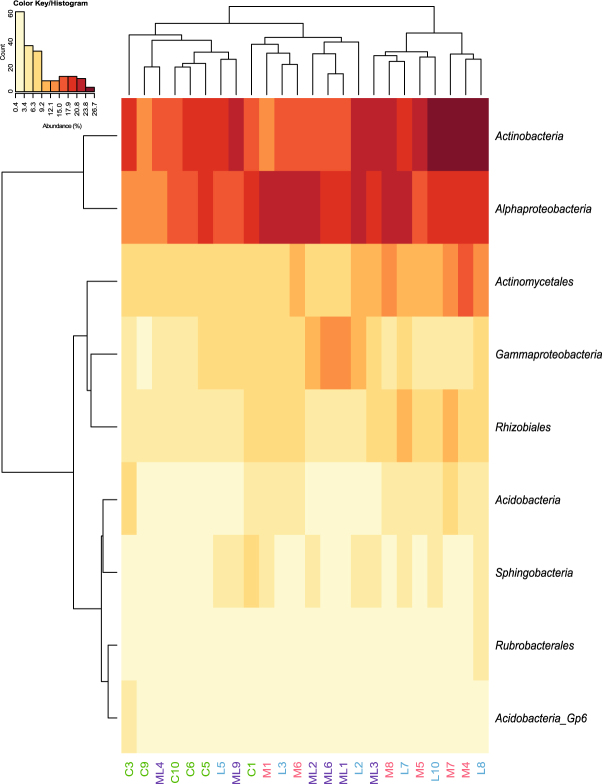
The abundance of several bacterial groups differed among soils in the different treatments. Heat-map analysis based on hierarchical clustering using the abundance of the main identified bacterial groups that showed significant differences by GLM among plant interaction treatments, *i.e*. soils without plants (control), with two *Maytenus* individuals (*Maytenus*-intraspecific), with two *Lycium* individuals (*Lycium*-intraspecific) or with one individual of each plant species (interspecific). Each grid unit in the heat-map is an individual value of abundance of a given bacterial group (rows) per sample (columns); warmer colors represent larger values of abundance and cooler colors represent lower values (see color key/histogram). C, control; M, *Maytenus*-intraspecific; L, *Lycium*-intraspecific and ML, interspecific. Numbers following letters denote replicates within each treatment.

Supporting our third hypothesis, the bacterial community in the interspecific treatment resembled the community of the most competitive plant species, *i.e. Lycium*. As such, the composition of the bacterial community in the interspecific treatment was more similar to soils with only *Lycium* than to soils with only *Maytenus* (Fig. 3, Supplementary Fig. S2 and Supplementary Table S4). When in monoculture, each plant species hosted a distinct soil bacterial community (Fig. 3), as expected. The variability in species composition of the community associated to *Lycium* was higher than that of *Maytenus* (Fig. 3). *Maytenus* promoted an increase in species richness compared to control soils with no plants (1037.2 ± 34.7 *vs*. 894.7 ± 32.1 operational taxonomic units (OTUs) per sample, respectively; Supplementary Table S2) and large modifications in community composition (Fig. 4 and Supplementary Table S5), similar to those reported for other facilitator species such as *Retama sphaerocarpa* ^13,14^. The dominant role of *Lycium* in defining bacterial community structure under interspecific competition could be modulated by the larger decrease in *Maytenus* root mass and root exudates^12^ when growing with *Lycium*. In addition, bacterial groups promoted by *Lycium* may have competitively displaced^29^ groups typically associated to *Maytenus*. Indeed, plant-driven impacts on particular soil microbial taxa may have cascading effects, resulting in altered interaction networks among soil taxa^27^.

Because *Maytenus* individuals seem to be associated with a specific bacterial community (Fig. 3), changes in the relative abundance of bacterial groups under interspecific competition could have also contributed to its poor performance. There is a need to know which microbial taxa are involved and the mechanisms by which they influence plant competition^18^. Changes are evident in our experiment (Fig. 4, see Supplementary Table S5 for a complete list) and concern, for instance, *Gammaproteobacteria*, a group that had higher abundance in the interspecific treatment than in *Maytenus* soils. This group includes many plant pathogens that could have negatively impacted *Maytenus*. Higher abundance was also observed for *Halomonas* and *Salinimicrobium*, genera that increase under drought conditions in semiarid environments^30^, and that could be related to greater water shortage in the interspecific treatment. *Rhizobiales* instead were less abundant in the interspecific treatment than in *Maytenus* soils, which, given their role on N fixation^14^, could have contributed to the lower N content and increased N competition in the interspecific treatment.

## Conclusions

The advent of powerful technologies to identify microbial communities is providing evidence on the widespread effects of microbes on ecological processes, more influential than acknowledged so far. Plant-plant interactions should be assessed not as isolated organisms in the environment but as a complex community of multiple trophic levels within which the soil microbiota is an important component^2,3^. Altogether, our results suggest that a major driver of the differences in plant performance between the two species was the ability of the better competitor (i.e. *Lycium*) to promote a preferred soil bacterial community. These data point to a novel mechanism controlling plant-plant competition and support the relevance of feedbacks between soil bacterial communities and plants for community dynamics. Since plant communities determine ecosystem properties, by influencing plant interactions the effect of microbes goes beyond a local process such as competition to impact ecosystem function and services.

## Methods

### Plant material, soil collection and experimental set-up

*Maytenus senegalensis* subsp. *europaea* (Celastraceae) is a thorny evergreen shrub up to 4 m tall with an intricate semispherical canopy. Many other plant species (shrubs, climbers, forbs, grasses) grow within its canopy forming patches with bare soil in between^31^. It is distributed throughout North Africa and Southeast Spain, where it is found in coastal areas^32^. *Lycium intricatum* (Solanaceae) is a thorny shrub with drought-deciduous, succulent leaves and shallow roots^33^ that can be found in coastal systems either associated to facilitator species such as *Maytenus* ^31^ or *Ziziphus lotus* or isolated^2^. In the field, *Lycium* seedlings may establish under the canopy of adult *Maytenus* shrubs^31^. Field observations of large dead *Maytenus* shrubs with large alive *Lycium* shrubs growing in their understorey (Hortal, personal observation) may suggest that *Lycium* could be able to outcompete *Maytenus* in the field. Shifts in the outcome of plant-plant interactions as seedlings become adults have been reported in many other systems^34,35^.

In March 2011, two-year old *Maytenus* and *Lycium* saplings were obtained from a local nursery (Rodalquilar, Almería, Spain). Root systems were carefully washed to remove excess of potting mix and saplings were planted in 6 L pots. Each pot contained two saplings of the same or different species; i.e., two *Maytenus* plants growing together (*Maytenus*-intraspecific), two *Lycium* plants (*Lycium*-intraspecific) or one *Maytenus* and one *Lycium* (interspecific). Control pots without plants were also established resulting in four treatments with 10 replicates each. Pots with only one individual were not included in the experimental design as we were interested on whether inter- and intraspecific competition affected soil bacterial communities in different ways, and not so much on the effect of intraspecific competition. Initial height and basal diameter were recorded for each sapling. Mean height was 29.34 ± 1.17 cm (mean ± standard error) and 33.14 ± 0.85 cm and mean diameter was 0.51 ± 0.03 cm and 0.59 ± 0.02 cm for *Maytenus* and *Lycium*, respectively, with no significant differences among treatments. Five additional *Maytenus* and *Lycium* individuals were randomly selected to establish initial dry mass after 48 h at 70 °C. Initial mean aboveground dry mass was 2.45 ± 0.28 g and 2.54 ± 0.30 g for *Maytenus* and *Lycium*, respectively. Root mass was 1.25 ± 0.18 g and 0.95 ± 0.06 g for *Maytenus* and *Lycium*, respectively.

Soil was obtained in March 2011 at Rambla del Toyo (Almería, Spain), a dry riverbed where both species co-exist forming typical coastal shrublands and where *Maytenus* and *Ziziphus lotus* are the dominant species. Soil from the first top 10 cm was collected from different bare areas within an approximately 2 ha plot, sieved through 4 mm mesh and mixed. It was sandy, poor in nutrients, with low water holding capacity and pH around 8–8.5^36^. Soil was used to fill the pots containing gravel at the bottom to improve aeration. Three soil samples were taken and stored at 4 °C for a maximum of 30 days^37^ to determine total N, total C and enzyme activities; additional samples were kept at −80 °C to perform molecular analysis. Total N and C were determined using an Elemental Analyzer (Leco Truspec, St. Joseph, MI, USA). Total N was 0.28 ± 0.03 g kg^−1^ and total C was 8.80 ± 0.18 g kg^−1^. Dehydrogenase activity was 5.00 ± 0.20 µg INTF g^−1^ soil h^−1^, phosphatase was 0.23 ± 0.01 μmol of p-nitrophenyl phosphate (PNP) g^−1^ h^−1^, urease was 0.37 ± 0.06 μmol N-NH_4_^+^ g^−1^ h^−1^ and β-glucosidase was 0.08 ± 0.01 μmol PNP g^−1^ h^−1^.

Pots were randomly distributed in a greenhouse at the Estación Experimental de Zonas Áridas in Almería (Spain) under natural temperature and irradiance regime. Mean annual temperature in the region is 18.8 °C with mean temperatures of 12.5 °C and 26.5 °C on the coldest and warmest months, respectively (www.meteodata.org/koppen). The experiment ran for one year, and for the length of the experiment pots were regularly watered, regularly shifted to avoid gradients, and plant mortality was recorded. To reduce the number of samples for analysis while keeping a reasonable number of replicates, six out of the 10 initial replicates per treatment were randomly selected for plant measurements and soil harvesting in March 2012, during the peak of plant productivity.

### Plant traits and growth measurements

Of these 6 replicates (pots with two plants), three replicates per treatment were selected for plant trait measurements following standard protocols^24^. At harvesting, three leaves per plant were sampled to measure specific leaf area (SLA) and three fine roots (<2 mm diameter, 10 cm in length approximately) per plant were collected to calculate specific root length (SRL). Mean values were obtained per each individual plant and used for statistical analyses. Plant height and stem diameter were also recorded at the end of the experiment. Shoot and root dry mass were weighted after drying at 70 °C for 48 h.

### Soil sampling and chemical analyses

At harvest, we sampled soil from the first top 10 cm between the two plants (4 cm distance from each plant) in each pot. Soil from control pots without plants was also collected. Each soil sample was homogenised and sieved through 2 mm mesh. Material was cleaned with ethanol 70% between samples. A total of 24 samples were collected, six per each plant interaction treatment (control -no plant-, *Maytenus*-intraspecific, *Lycium*-intraspecific, and interspecific). Each soil sample was divided into two subsamples, one was stored at −80 °C for molecular analyses and the other was kept at 4 °C for a maximum of 30 days^37^ for chemical and enzymatic activity analyses. Available NH_4_^+^ and total organic C (TOC) were determined using an Elemental Analyzer (Leco Truspec, St. Joseph, MI, USA). Concentration of available NH_4_^+^ in soil was measured using a 1 M KCl solution, in a ratio 1:10 soil:solution, to extract the available fraction of this cation and then determined by a colorimetric method^38^. Three grams of soil per sample were dried at 105 °C for 24 hours and weighed to analyze soil moisture.

### Soil enzyme activities

Soil dehydrogenase activity was determined in 1 g of soil incubated with 0.2 ml of p-iodonitrotetrazolium chloride (INT, 0.4% w/v) at room temperature for 20 hours. The reduction of INT to p-iodonitrotetrazolium formazan (INTF) at soil pH was estimated by a modification of a reported protocol^39^. The INTF produced was extracted with 10 ml of methanol, and the absorbance of the filtrate was measured in a spectrophotometer (Helios Alpha, Thermo, UK) at 490 nm. The β-glucosidase and alkaline phosphatase activities were determined by following standard methods^40,41^. Two millilitres of MUB (Modified Universal Buffer), pH 6.5 for the β-glucosidase assay and pH 11 for the alkaline phosphatase assay, and 0.5 ml of p-nitrophenyl substrate (p-nitrophenyl-β-d-glucopyranoside for β-glucosidase and p-nitrophenyl phosphate for alkaline phosphatase) were added to 0.5 g of soil. The mixtures were incubated at 37 °C for 1 h. Then, the p-nitrophenol released was measured by colorimetry in a UV-vis spectrophotometer (Helios Alpha, Thermo, UK) at 400 nm. The urease activity was determined as a previously reported method^42^. In this procedure, 0.5 ml of a solution of urea (0.48%) and 4 ml of borate buffer (pH 10) were added to 1 g of soil (0.4 g of compost) and then incubated for 2 h at 37 °C. The ammonium concentration of the centrifuged extracts was determined by a modified indophenol-blue reaction and measured at 690 nm by spectrophotometry.

### Soil bacterial community composition: 16S rDNA pyrosequencing

DNA was extracted from 0.25 g of homogenised soil of each of the 24 samples using the PowerSoil^®^ DNA Isolation Kit (MO BIO Laboratories, Inc., Carlsbad, USA) following manufacturer’s directions. A 16S rDNA gene fragment corresponding to V1 and V2 regions was amplified^19^. PCR amplifications were performed in 50 μl reaction volumes containing ultrapure H_2_O, 2.5 × 5 PRIME MasterMix including 1.5 mM Magnesium, 200 μM dNTPs, 1.25 U Taq polymerase (5 PRIME, Hamburg, Germany), 0.2 μM of primers and 5–10 ng of template DNA. Fragments were amplified under the following conditions: initial denaturation at 94 °C for 3 minutes, followed by 30 cycles with denaturation at 94 °C for 40 seconds, annealing at 52 °C for 40 seconds and extension at 68 °C for 35 seconds, with a final extension at 68 °C for 7 minutes. Each sample was amplified in triplicate, pooled and purified using the QIAquick^®^ PCR Purification Kit (Qiagen, Hilden, Germany). Amplification was checked by electrophoresis in 2% agarose gels stained with SYBR^®^ Safe DNA Gel Stain (Invitrogen™, Carlsbad, USA) and bands were visualized using UV light in a Gel Doc™ EZ Imager (BIO-RAD, Hercules, USA).

DNA concentration of each purified sample was determined as the mean of three lectures in NanoDrop 2000c (Thermo Scientific, Wilmington, USA). Equal amounts of PCR product for each sample were combined in a single tube to obtain an equimolar pool. The pool was pyrosequenced in a Roche Genome Sequencer FLX System (Roche, Basel, Switzerland) using 454 Titanium chemistry at Lifesequencing (Valencia, Spain).

### Processing of pyrosequencing data

The 16 S rDNA sequence data were processed using mothur v.1.32.1^43,44^. Sequences were denoised using PyroNoise implemented in the mothur command shhh.flows. Sequences with more than 1 mismatch to the barcode, two mismatches to the primer or homopolymers >8 bp were removed from the dataset. Sequences were trimmed for primers and assigned to samples according to their barcode. Mean length of retained sequences was 269 bp. Sequences were aligned using the SILVA database. To further reduce sequencing errors, sequences that were within 2 bp of a more abundant sequence were merged using the pre.cluster command. Chimeras were identified using UCHIME with the sequences as their own reference and removed from the dataset. Sequences were classified using the mothur version of the RDP Bayesian classifier^45^. Any sequences classified as Mitochondria, Chloroplast, Archaea, Eukaryota or unknown (i.e. not classified at the Kingdom level) were removed from the dataset. Aligned sequences were clustered into operational taxonomic units (OTUs) defined at 97% similarity cutoff using the average neighbour method. The consensus taxonomy per each OTU was obtained. The number of sequences in each sample was normalized to 2968 (corresponding to the sample with the fewest sequences). We used Past software version 2.12^46^ to calculate the richness, Shannon diversity index (H’) and evenness for each sample. Relative abundances of the different taxonomic groups in each sample were calculated.

### Statistical analyses

Differences among treatments in shoot and root biomass, SLA and SRL were analyzed with general linear models (GLM) full factorial design including species (*Maytenus*, *Lycium*), plant-plant interaction (intra- *vs*. interspecific) as fixed factors and the statistical interaction among these factors. Differences in soil TOC, available NH_4_^+^, enzyme activities, relative abundance of the different taxa, OTU richness and Shannon’s diversity index were analyzed with GLMs that included plant interaction treatment (control, *Maytenus*-intraspecific, *Lycium*-intraspecific and interspecific) as fixed factor. Because in three pots of the interspecific treatment the *Maytenus* individual died before harvest, we repeated the same analyses after excluding those three replicates to ensure results were the same and thus were not explained by the death of *Maytenus* individuals. Moreover, deaths occurred towards the end of the experiment and were unrelated to transplant; therefore, those *Maytenus* individuals were alive for most of the experiment. We selected a variance function structure with different coefficients of the variance function for different strata to avoid heteroscedasticity (varIdent R function)^47^ and a compound symmetry as the spatial autocorrelation structure function (as two plant individuals grew in the same pot). Post-hoc comparisons were performed using the Fisher’s LSD test. Differences in mortality among treatments were assessed with generalized models. Similarity in OTUs composition among treatments was analysed with Non-metric Multidimensional Scaling (NMDS) analysis using Bray-Curtis similarity index and one-way NPMANOVA with 9999 permutations in Past^46^. The abundance of the main identified bacterial groups showing significant differences among treatments by GLM was used in a heat-map analysis based on hierarchical clustering using the average linkage method with Euclidean distance and bootstrap with 500 iterations^48^. Statistical analyses were done with R^49^ using the interface implemented in InfoStat statistical software^50^. Results are presented as mean values ± 1 SE throughout the text. Differences among treatments were considered significant at p < 0.05.

### Data availability

Pyrosequencing data are deposited in the European Nucleotide Archive (ENA) under the accession number PRJEB22755.

## Electronic supplementary material

Supplementary Information
