# Supplementary material for: Plant-plant competition outcomes are modulated by plant effects on the soil bacterial community
Source: Sci Rep. 2017 Dec 19;7:17756. doi: 10.1038/s41598-017-18103-5 (PMC5736699; doi:10.1038/s41598-017-18103-5)
Supplement: Supplementary file 1 — Supplementary Information [file 41598_2017_18103_MOESM1_ESM.pdf]

**Plant-plant competition outcomes are modulated by plant effects on the soil bacterial community**

***Supplementary Information (Scientific Reports)***

Hortal S, Lozano YM, Bastida F, Armas C, Moreno J.L., Garcia C, Pugnaire FI

**Contents:**

Supplementary Figures S1 and S2.

Supplementary Tables S1 to S5.

Supplementary Figure S1. The presence of *Lycium* induced increased mortality in *Maytenus*. Percentage of mortality in *Maytenus* and *Lycium* plants growing under intra- or interspecific interaction. Different letters in a graph indicate significant differences ($p<0.05$) among treatments. n=10

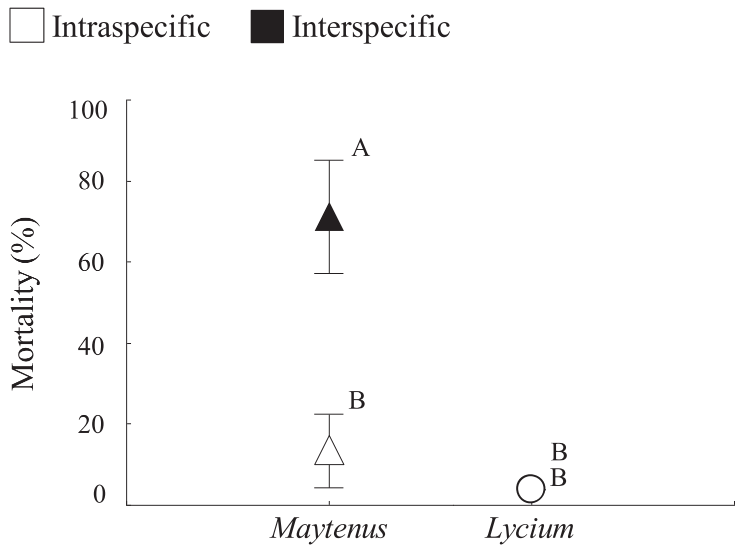

Supplementary Figure S2. Similarity between the bacterial community in the interspecific treatment and in *Lycium* soils was not due to *Maytenus* mortality. Ordination of soil bacterial community composition by Non-metric multidimensional scaling (NMDS) when excluding the three interspecific pots in which the *Maytenus* individual died before harvest was similar to the NMDS including all samples (Fig.3). Samples are coded by plant interaction treatment, in particular: asterisks = control (soils without plants), triangles = *Maytenus*-intraspecific (soils with two *Maytenus* individuals), circles = *Lycium*-intraspecific (soils with two *Lycium* individuals); black filled squares = interspecific (soils with one individual of each plant species); n=6 for all treatments except n=3 for interspecific. Stress = 0.2.

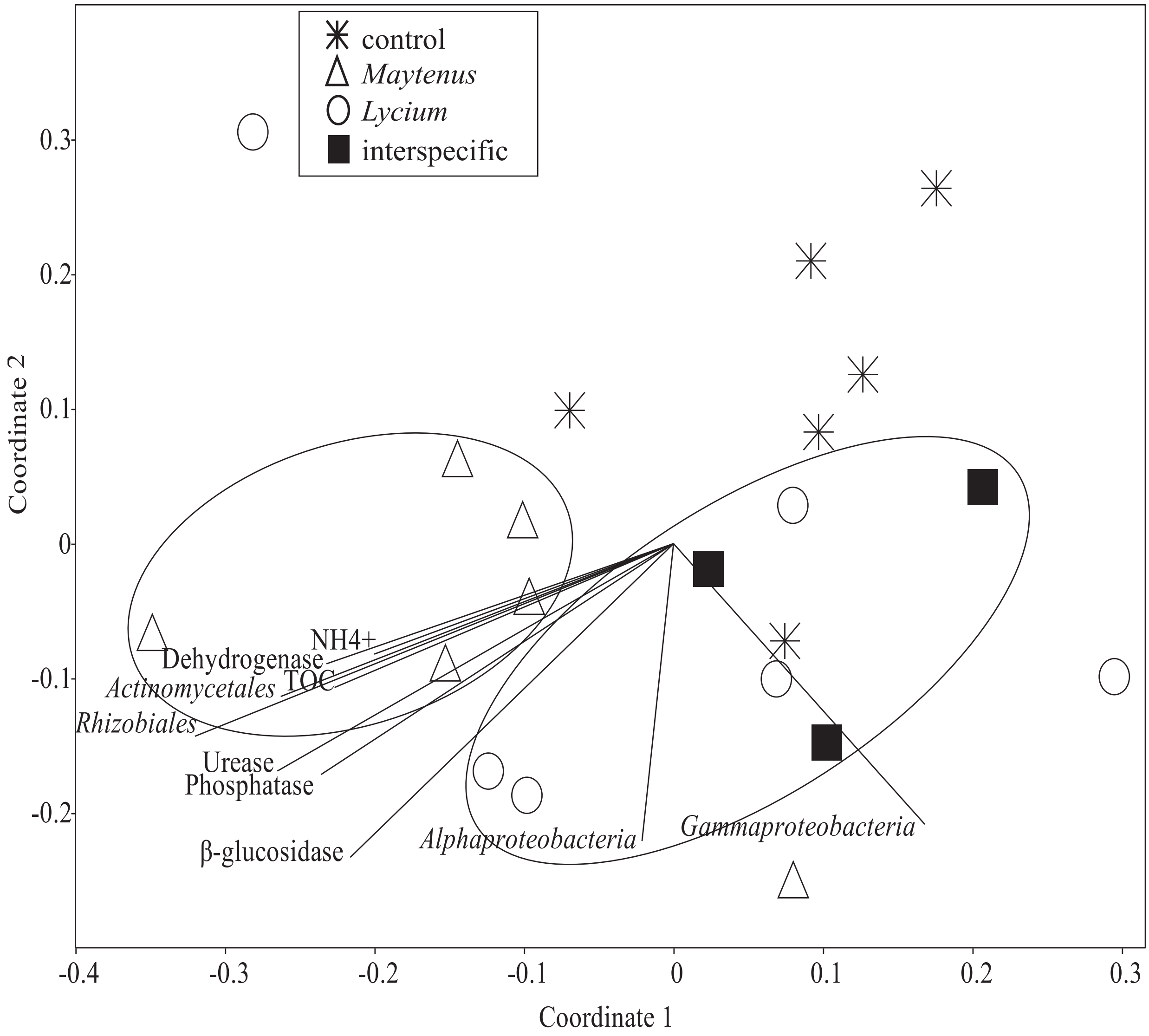

Supplementary Table S1. Results from *a priori* contrasts tests between interaction treatments (intraspecific *vs* interspecific) on plant mortality for each plant species (*Maytenus*, *Lycium*). Significant differences between contrasted treatments are marked in bold.

| **Plant mortality**<br>(inter *vs* intraspecific interaction) | **Chi-square** | **p** |
|---|---|---|
| *Lycium* | 0.00 | >0.99 |
| *Maytenus* | 5.84 | **0.01** |

Supplementary Table S2. Results from general linear models on a) plant traits and b) soil properties. Traits were measured in individuals of each plant species (*Maytenus, Lycium*) under each interaction type (intraspecific, interspecific). Soil properties were measured in pots of the four plant interaction treatments, *i.e.* soils without plants (control), with two *Maytenus* individuals (*Maytenus*-intraspecific), with two *Lycium* individuals (*Lycium*-intraspecific) or with one individual of each plant species (interspecific). Results show F values and its significance (*, **, ***, at p < 0.05, 0.01, 0.001, respectively; n.s. = non significant). Significant values are marked in bold.

a)

| **Plant traits** | **Species (Sp)** | **Interaction (I)** | **Sp x I** |
|---|---|---|---|
| Shoot biomass (g) | $2.93^{n.s.}$ | **20.65*** ** | **6.34** ** |
| Root biomass (g) | $0.82^{n.s.}$ | **17.51*** ** | $4.07^{n.s}$ |
| SLA ($m^2$ $kg^{-1}$) | **126.86*** ** | $0.35^{n.s}$ | **10.84*** ** |
| SRL (m $g^{-1}$) | $1.14^{n.s}$ | $2.38^{n.s}$ | **6.67** ** |

b)

| **Soil properties** | **Plant interaction** |
|---|---|
| Soil moisture (%) | $1.07^{n.s}$ |
| TOC (%) | $2.15^{n.s}$ |
| Available $NH_4^+$ (mg $g^{-1}$) | **28.04*** ** |
| Dehydrogenase (µg INTF $g^{-1}$ $h^{-1}$) | **36.40*** ** |
| β–glucosidase (µmol PNP $g^{-1}$ $h^{-1}$) | **36.32*** ** |
| Phosphatase (µmol PNP $g^{-1}$ $h^{-1}$) | **16.78*** ** |
| Urease (µmol N-$NH_4^+$ $g^{-1}$ $h^{-1}$) | **11.29*** ** |
| Bacterial richness | **3.78** ** |
| Bacterial diversity | $2.39^{n.s}$ |

Supplementary Table S3. Mean enzyme activities (±1 SE) in soils of the different plant interaction treatments, *i.e.* soils without plants (control), with two *Maytenus* individuals (*Maytenus*-intraspecific), with two *Lycium* individuals (*Lycium*-intraspecific) or with one individual of each plant species (interspecific), after excluding the three pots of the interspecific treatment in which the *Maytenus* individual died before harvest. Different letters in a row indicate significant differences ($p < 0.05$) among treatments after Fisher's LSD test; n= 6 for all treatments except n=3 for interspecific. Last column shows F values of the performed general linear model and its significance (*** at $p < 0.001$). Significant values are marked in bold. *Maytenus* = *Maytenus*-intraspecific; *Lycium* = *Lycium*-intraspecific. See units in Supplementary Table S2.

| **Enzyme** | **Control** | ***Maytenus*** | ***Lycium*** | **Interspecific** | **F value** |
|---|---|---|---|---|---|
| Dehydrogenase | 2.69 ± 0.15 b | 4.07 ± 0.15 a | 2.25 ± 0.15 b | 2.18 ± 0.21 b | **31.41***** |
| β-glucosidase | 0.05 ± 0.002 c | 0.14 ± 0.01 a | 0.13 ± 0.01 a | 0.09 ± 0.01 b | **70.71***** |
| Phosphatase | 0.24 ± 0.02 b | 0.42 ± 0.02 a | 0.44 ± 0.02 a | 0.28 ± 0.03 b | **17.85***** |
| Urease | 0.30 ± 0.03 b | 0.46 ± 0.03 a | 0.46 ± 0.03 a | 0.33 ± 0.04 b | **8.84***** |

Supplementary Table S4. *F* values for pairwise comparisons of soil bacterial community composition between plant interaction treatments, *i.e.* soils without plants (control), with two *Maytenus* individuals (*Maytenus*-intraspecific), with two *Lycium* individuals (*Lycium*-intraspecific) or with one individual of each plant species (interspecific), using one-way NPMANOVA analysis with Bray-Curtis similarity index. Analysis was performed including all samples (left) and excluding the three pots from the interspecific treatment in which the *Maytenus* individual died before harvest (right). *Maytenus* = *Maytenus*-intraspecific; *Lycium* = *Lycium*-intraspecific. Results show F values and its significance (*, ** at p < 0.05, 0.01 respectively; *n.s.* = non significant); n=9999 permutations.

| | **All samples** | | | **Excluding 3 pots** | | |
|---|---|---|---|---|---|---|
| **Treatment** | Control | *Maytenus* | *Lycium* | Control | *Maytenus* | *Lycium* |
| Control | | | | | | |
| *Maytenus* | **1.78**** | | | **1.78**** | | |
| *Lycium* | **1.46*** | **1.34**** | | **1.46*** | **1.34**** | |
| Interspecific | **1.34*** | **1.73**** | $1.15^{n.s}$ | **1.35*** | **1.42*** | $0.99^{n.s}$ |

Supplementary Table S5. Mean relative abundance (± SE) of the identified bacterial taxonomic groups in the different plant interaction treatments, *i.e.* soils without plants (control), with two *Maytenus* individuals (*Maytenus*-intraspecific), with two *Lycium* individuals (*Lycium*-intraspecific) or with one individual of each plant species (interspecific). Taxonomic levels for each group are shown in the first column. Different letters in a row indicate significant differences ($p<0.05$) among treatments by Fisher post-hoc comparison; n=6. Groups showing differences among treatments are marked in bold. *Maytenus* = *Maytenus*-intraspecific; *Lycium* = *Lycium*-intraspecific.

| | **Bacterial Group** | **Control** | ***Maytenus*** | ***Lycium*** | **Interspecific** |
|---|---|---|---|---|---|
| **Phylum** | ***Acidobacteria*** | **3.39 ± 0.52 a** | **4.40 ± 0.52 a** | **3.75 ± 0.52 a** | **2.22 ± 0.52 b** |
| Phylum | *Actinobacteria* | 17.50 ± 1.41 | 21.20 ± 1.41 | 21.32 ± 1.41 | 18.29 ± 1.41 |
| Phylum | *Armatimonadetes* | 0.06 ± 0.05 | 0.11 ± 0.05 | 0.06 ± 0.05 | 0.11 ± 0.05 |
| Phylum | *Bacteroidetes* | 13.36 ± 1.42 | 10.61 ± 1.42 | 13.03 ± 1.42 | 14.16 ± 1.42 |
| Phylum | *Chloroflexi* | 0.12 ± 0.08 | 0.27 ± 0.08 | 0.05 ± 0.08 | 0.1 ± 0.08 |
| Phylum | *Deinococcus* | 0.04 ± 0.04 | 0.08 ± 0.05 | 0.14 ± 0.05 | 0.06 ± 0.05 |
| Phylum | *Firmicutes* | 1.67 ± 0.23 | 1.93 ± 0.23 | 1.49 ± 0.23 | 2.03 ± 0.23 |
| **Phylum** | ***Gemmatimonadetes*** | **1.36 ± 0.16 a** | **1.20 ± 0.16 a** | **0.67 ± 0.16 b** | **0.88 ± 0.16 b** |
| Phylum | *Nitrospira* | 0.21 ± 0.07 | 0.34 ± 0.07 | 0.25 ± 0.07 | 0.21 ± 0.07 |
| Phylum | *OD1* | 0.01 ± 0.02 | 0.02 ± 0.02 | 0 ± 0.02 | 0.03 ± 0.02 |
| Phylum | *Planctomycetes* | 0.08 ± 0.02 a | 0.06 ± 0.02 | 0.03 ± 0.02 | 0.01 ± 0.02 |
| **Phylum** | ***Proteobacteria*** | **25.50 ± 1.66 b** | **30.52 ± 1.66 a** | **30.78 ± 1.66 a** | **30.75 ± 1.66 a** |
| Phylum | *TM7* | 0.50 ± 0.18 | 0.35 ± 0.18 | 0.84 ± 0.18 | 0.87 ± 0.18 |
| Class | *Acidobacteria_GP1* | 0.04 ± 0.02 | 0.02 ± 0.02 | 0.04 ± 0.02 | 0.02 ± 0.02 |
| Class | *Acidobacteria_Gp16* | 0.23 ± 0.08 | 0.38 ± 0.08 | 0.49 ± 0.08 | 0.47 ± 0.08 |
| **Class** | ***Acidobacteria_Gp3*** | **0.38 ± 0.16 a** | **0.35 ± 0.05 a** | **0.27 ± 0.10 a** | **0.11 ± 0.04 b** |
| **Class** | ***Acidobacteria_Gp4*** | **0.67 ± 0.23 b** | **1.82 ± 0.23 a** | **1.33 ± 0.23 a** | **0.67 ± 0.23 b** |
| **Class** | ***Acidobacteria_Gp6*** | **1.87 ± 0.31 a** | **1.69 ± 0.31 a** | **1.40 ± 0.31 a** | **0.85 ± 0.31 b** |
| Class | *Acidobacteria_Gp7* | 0.07 ± 0.04 | 0.05 ± 0.04 | 0.11 ± 0.04 | 0.01 ± 0.04 |
| **Class** | ***Actinobacteria*** | **17.33 ± 1.42 b** | **21.04 ± 1.42 a** | **21.22 ± 1.42 a** | **18.15 ± 1.42 b** |
| **Class** | ***Alphaproteobacteria*** | **16.74 ± 0.95 b** | **20.33 ± 0.95 a** | **20.23 ± 0.95 a** | **18.47 ± 0.95 b** |
| Class | *Armatimonadetes_GP4* | 0.02 ± 0.04 | 0.02 ± 0.04 | 0.01 ± 0.04 | 0.1 ± 0.04 |
| Class | *Armatimonadia* | 0.04 ± 0.03 | 0.06 ± 0.03 | 0.03 ± 0.03 | 0 ± 0.03 |
| Class | *Bacilli* | 1.62 ± 0.21 | 1.84 ± 0.21 | 1.41 ± 0.21 | 1.83 ± 0.21 |
| Class | *Bacteroidetes_incertae* | 0.02 ± 0.03 | 0.08 ± 0.03 | 0.05 ± 0.03 | 0.02 ± 0.03 |
| Class | *Betaproteobacteria* | 1.02 ± 0.26 | 1.46 ± 0.26 | 1.20 ± 0.26 | 1.19 ± 0.26 |

| | | | | | |
|---|---|---|---|---|---|
| Class | *Chloroflexi* | 0.02 ± 0.02 | 0.06 ± 0.02 | 0.01 ± 0.02 | 0.03 ± 0.02 |
| Class | *Deinococci* | 0.04 ± 0.05 | 0.08 ± 0.05 | 0.14 ± 0.05 | 0.06 ± 0.05 |
| Class | *Deltaproteobacteria* | 1.16 ± 0.26 | 0.96 ± 0.31 | 0.55 ± 0.08 | 0.63 ± 0.05 |
| Class | *Flavobacteria* | 7.46 ± 0.90 | 5.91 ± 0.90 | 6.55 ± 0.90 | 9.00 ± 0.90 |
| **Class** | ***Gammaproteobacteria*** | **5.37 ± 0.80 b** | **5.96 ± 0.80 b** | **7.70 ± 0.80 a** | **9.16 ± 0.79 a** |
| **Class** | ***Gemmatimonadetes*** | **1.34 ± 0.16 a** | **1.19 ± 0.16 a** | **0.67 ± 0.16 b** | **0.86 ± 0.16 b** |
| Class | *Nitrospira* | 0.21 ± 0.06 | 0.34 ± 0.06 | 0.25 ± 0.07 | 0.21 ± 0.07 |
| **Class** | ***Sphingobacteria*** | **3.13 ± 0.82 b** | **2.76 ± 0.25 b** | **4.09 ± 0.37 a** | **3.09 ± 0.30 b** |
| **Class** | ***TM7*** | **0.50 ± 0.18 b** | **0.35 ± 0.18 b** | **0.84 ± 0.18 a** | **0.87 ± 0.18 a** |
| Order | *Acidimicrobiales* | 2.72 ± 0.32 | 2.22 ± 0.32 | 2.99 ± 0.32 | 2.69 ± 0.32 |
| Order | *Acidobacteria_Gp1* | 0.04 ± 0.02 | 0.02 ± 0.01 | 0.04 ± 0.02 | 0.02 ± 0.02 |
| Order | *Acidobacteria_Gp16* | 0.23 ± 0.08 | 0.38 ± 0.08 | 0.49 ± 0.08 | 0.47 ± 0.08 |
| **Order** | ***Acidobacteria_GP3*** | **0.38 ± 0.16 a** | **0.35 ± 0.05 a** | **0.27 ± 0.09 a** | **0.11 ± 0.04 b** |
| **Order** | ***Acidobacteria_Gp4*** | **0.67 ± 0.20 b** | **1.82 ± 0.30 a** | **1.33 ± 0.24 a** | **0.67 ± 0.16 b** |
| **Order** | ***Acidobacteria_Gp6*** | **1.91 ± 0.55 a** | **1.71 ± 0.20 a** | **1.42 ± 0.21 a** | **0.86 ± 0.10 b** |
| **Order** | ***Acidobacteria-Gp7*** | **0.07 ± 0.04 a** | **0.05 ± 0.04 a** | **0.11 ± 0.04 a** | **0.005 ± 0.04 b** |
| **Order** | ***Actinomycetales*** | **7.24 ± 0.84 b** | **11.75 ± 0.83 a** | **9.92 ± 0.8 a** | **8.46 ± 0.84 b** |
| Order | *Alphaproteobacteria* | 0.06 ± 0.04 | 0.11 ± 0.04 | 0.1 ± 0.04 | 0.1 ± 0.04 |
| Order | *Alteromonadales* | 0.36 ± 0.11 | 0.39 ± 0.14 | 0.49 ± 0.11 | 0.64 ± 0.16 |
| Order | *Armatimonadales* | 0.04 ± 0.03 | 0.06 ± 0.03 | 0.03 ± 0.03 | 0.00 ± 0.03 |
| Order | *Armatimonadetes_Gp4* | 0.02 ± 0.04 | 0.02 ± 0.04 | 0.01 ± 0.04 | 0.1 ± 0.04 |
| Order | *Bacillales* | 1.65 ± 0.21 | 1.85 ± 0.21 | 1.41 ± 0.21 | 1.83 ± 0.21 |
| Order | *Bacteroidetes_incertae* | 0.02 ± 0.03 | 0.08 ± 0.03 | 0.05 ± 0.03 | 0.02 ± 0.03 |
| Order | *Burkholderiales* | 0.53 ± 0.18 | 0.96 ± 0.18 | 0.59 ± 0.18 | 0.71 ± 0.18 |
| Order | *Caulobacterales* | 0.11 ± 0.05 | 0.18 ± 0.05 | 0.06 ± 0.02 | 0.07 ± 0.02 |
| Order | *Chloroflexales* | 0.02 ± 0.02 | 0.06 ± 0.02 | 0.01 ± 0.02 | 0.02 ± 0.02 |
| Order | *Chromatiales* | 0.28 ± 0.14 | 0.28 ± 0.14 | 0.69 ± 0.14 | 0.27 ± 0.14 |
| **Order** | ***Clostridiales*** | **0.02 ± 0.01 b** | **0.04 ± 0.01 a** | **0.05 ± 0.01 a** | **0.06 ± 0.01 a** |
| Order | *Deinococcales* | 0.04 ± 0.05 | 0.08 ± 0.05 | 0.14 ± 0.05 | 0.06 ± 0.05 |
| Order | *Euzebyales* | 0.27 ± 0.08 | 0.16 ± 0.08 | 0.35 ± 0.08 | 0.39 ± 0.08 |
| Order | *Flavobacteriales* | 7.47 ± 0.90 | 5.91 ± 0.90 | 6.58 ± 0.90 | 9.00 ± 0.90 |
| Order | *Gemmatimonadales* | 1.36 ± 0.16 a | 1.20 ± 0.16 a | 0.67 ± 0.16 b | 0.88 ± 0.16 b |
| Order | *Legionellales* | 0.02 ± 0.02 | 0.11 ± 0.02 | 0.06 ± 0.02 | 0.03 ± 0.02 |
| Order | *Myxococcales* | 1.07 ± 0.25 | 0.82 ± 0.30 | 0.41 ± 0.03 | 0.43 ± 0.03 |
| Order | *Nitriliruptorales* | 0.12 ± 0.07 | 0.00 ± 0.07 | 0.06 ± 0.07 | 0.25 ± 0.07 |
| Order | *Nitrospirales* | 0.21 ± 0.07 | 0.34 ± 0.07 | 0.25 ± 0.07 | 0.21 ± 0.07 |
| Order | *Oceanospirillales* | 0.44 ± 0.25 | 0.49 ± 0.25 | 0.37 ± 0.25 | 1.15 ± 0.25 |
| **Order** | ***Planctomycetales*** | **0.07 ± 0.02 a** | **0.06 ± 0.03 a** | **0.03 ± 0.01 a** | **0.01 ± 0.01 b** |
| Order | *Pseudomonadales* | 0.34 ± 0.12 | 0.32 ± 0.12 | 0.58 ± 0.12 | 0.62 ± 0.12 |
| **Order** | ***Rhizobiales*** | **5.55 ± 0.56 b** | **8.48 ± 0.56 a** | **6.86 ± 0.56 a** | **5.07 ± 0.56 b** |
| Order | *Rhodobacterales* | 5.31 ± 0.78 | 5.30 ± 0.78 | 5.38 ± 0.78 | 7.23 ± 0.78 |
| Order | *Rhodospirillales* | 0.59 ± 0.14 | 0.89 ± 0.14 | 1.01 ± 0.14 | 0.52 ± 0.14 |
| **Order** | ***Rubrobacterales*** | **2.35 ± 0.21 a** | **2.26 ± 0.21 a** | **2.36 ± 0.21 a** | **1.81 ± 0.21 b** |

| | | | | | |
|---|---|---|---|---|---|
| **Order** | ***Solirubrobacterales*** | **1.86 ± 0.26 a** | **2.10 ± 0.26 a** | **1.96 ± 0.26 a** | **1.55 ± 0.26 b** |
| Order | *Sphingobacteriales* | 3.18 ± 0.84 | 2.81 ± 0.25 | 4.10 ± 0.36 | 3.12 ± 0.29 |
| Order | *Sphingomonadales* | 4.29 ± 0.48 | 3.65 ± 0.48 | 5.44 ± 0.48 | 4.44 ± 0.48 |
| Order | *Thermoleophilales* | 0.02 ± 0.03 | 0.07 ± 0.03 | 0.06 ± 0.03 | 0.1 ± 0.03 |
| Order | *Thiotrichales* | 0.23 ± 0.06 | 0.13 ± 0.06 | 0.08 ± 0.06 | 0.22 ± 0.06 |
| Order | *TM7_order* | 0.50 ± 0.18 | 0.35 ± 0.18 | 0.84 ± 0.18 | 0.87 ± 0.18 |
| Order | *Xanthomonadales* | 0.49 ± 0.12 | 0.85 ± 0.12 | 0.88 ± 0.12 | 0.66 ± 0.12 |
| **Genus** | ***Aciditerrimonas*** | **0.71 ± 0.20 a** | **0.13 ± 0.04 b** | **0.50 ± 0.37 a** | **0.17 ± 0.05 b** |
| Genus | *Adhaeribacter* | 0.02 ± 0.06 | 0.06 ± 0.06 | 0.15 ± 0.06 | 0.05 ± 0.06 |
| **Genus** | ***Aeromicrobium*** | **0.005 ± 0.02 b** | **0.07 ± 0.02 a** | **0.06 ± 0.02 a** | **0.07 ± 0.02 a** |
| Genus | *Agromyces* | 0.02 ± 0.04 | 0.13 ± 0.04 | 0.1 ± 0.04 | 0.03 ± 0.04 |
| Genus | *Algoriphagus* | 0.02 ± 0.04 | 0.03 ± 0.04 | 0.1 ± 0.04 | 0.08 ± 0.04 |
| Genus | *Altererythrobacter* | 0.98 ± 0.21 | 0.63 ± 0.21 | 0.72 ± 0.21 | 0.54 ± 0.21 |
| Genus | *Alteromonadales* | 0.36 ± 0.11 | 0.39 ± 0.14 | 0.49 ± 0.11 | 0.64 ± 0.16 |
| Genus | *Armatimonadetes_gp4* | 0.02 ± 0.04 | 0.02 ± 0.04 | 0.01 ± 0.04 | 0.1 ± 0.04 |
| Genus | *Armatimonas* | 0.04 ± 0.03 | 0.06 ± 0.03 | 0.03 ± 0.03 | 0 ± 0.03 |
| Genus | *Arthrobacter* | 0.53 ± 0.15 | 0.53 ± 0.15 | 0.82 ± 0.15 | 0.71 ± 0.15 |
| Genus | *Aurantimonas* | 0.16 ± 0.04 | 0.20 ± 0.14 | 0.15 ± 0.02 | 0.09 ± 0.05 |
| Genus | *Bacillus-genus* | 0.90 ± 0.12 | 0.97 ± 0.12 | 0.68 ± 0.12 | 0.64 ± 0.12 |
| Genus | *Blastococcus* | 0.23 ± 0.05 | 0.30 ± 0.05 | 0.30 ± 0.05 | 0.28 ± 0.05 |
| **Genus** | ***Brevundimonas*** | **0.10 ± 0.02 a** | **0.09 ± 0.02 a** | **0.02 ± 0.02 b** | **0.04 ± 0.02 b** |
| **Genus** | ***Cellvibrio*** | **0.05 ± 0.02 b** | **0.09 ± 0.03 b** | **0.27 ± 0.09 a** | **0.26 ± 0.16 a** |
| Genus | *Citreimonas* | 0.06 ± 0.04 | 0.07 ± 0.04 | 0.07 ± 0.04 | 0.11 ± 0.04 |
| Genus | *Conexibacter* | 0.05 ± 0.01 | 0.08 ± 0.03 | 0.06 ± 0.02 | 0.02 ± 0.01 |
| **Genus** | ***Croceicoccus*** | **0.06 ± 0.02 a** | **0.01 ± 0.01 b** | **0.06 ± 0.02 a** | **0.05 ± 0.02 a** |
| **Genus** | ***Devosia*** | **0.21 ± 0.05 b** | **0.16 ± 0.05 b** | **0.39 ± 0.05 a** | **0.24 ± 0.05 b** |
| Genus | *Dongia* | 0.07 ± 0.04 | 0.05 ± 0.01 | 0.11 ± 0.06 | 0.09 ± 0.04 |
| **Genus** | ***Ensifer*** | **0.08 ± 0.02 b** | **0.17 ± 0.04 a** | **0.06 ± 0.02 b** | **0.11 ± 0.03 b** |
| Genus | *Erythrobacter* | 0.80 ± 0.42 | 0.44 ± 0.13 | 0.29 ± 0.10 | 0.31 ± 0.10 |
| Genus | *Euzebya* | 0.27 ± 0.08 | 0.16 ± 0.08 | 0.35 ± 0.08 | 0.39 ± 0.08 |
| **Genus** | ***Flavisolibacter*** | **0.08 ± 0.03 a** | **0.07 ± 0.03 a** | **0.08 ± 0.03 a** | **0.02 ± 0.02 b** |
| Genus | *Fulvimarina* | 0.04 ± 0.06 | 0.08 ± 0.06 | 0.06 ± 0.06 | 0.12 ± 0.06 |
| Genus | *Geminicoccus* | 0.06 ± 0.04 | 0.11 ± 0.04 | 0.1 ± 0.04 | 0.1 ± 0.04 |
| **Genus** | ***Gemmatimonas*** | **1.36 ± 0.16 a** | **1.20 ± 0.16 a** | **0.67 ± 0.16 b** | **0.88 ± 0.16 b** |
| Genus | *Geodermatophilus* | 0.11 ± 0.06 | 0.05 ± 0.02 | 0.06 ± 0.01 | 0.04 ± 0.01 |
| Genus | *Gp1* | 0.04 ± 0.02 | 0.02 ± 0.02 | 0.04 ± 0.02 | 0.02 ± 0.02 |
| Genus | *Gp16* | 0.23 ± 0.08 | 0.38 ± 0.08 | 0.49 ± 0.08 | 0.47 ± 0.08 |
| **Genus** | ***Gp3*** | **0.38 ± 0.16 a** | **0.35 ± 0.05 a** | **0.27 ± 0.09 a** | **0.11 ± 0.04 b** |
| **Genus** | ***Gp4*** | **0.67 ± 0.20 b** | **1.82 ± 0.29 a** | **1.33 ± 0.24 a** | **0.67 ± 0.17 b** |
| **Genus** | ***Gp6*** | **1.91 ± 0.55 a** | **1.71 ± 0.20 a** | **1.42 ± 0.21 a** | **0.86 ± 0.10 b** |
| **Genus** | ***Gp7*** | **0.07 ± 0.03 a** | **0.05 ± 0.02 a** | **0.11 ± 0.05 a** | **0.005 ± 0.005 b** |
| Genus | *Gramella* | 0.16 ± 0.14 | 0.22 ± 0.14 | 0.06 ± 0.14 | 0.2 ± 0.14 |
| **Genus** | ***Hahella*** | **0.09 ± 0.04 a** | **0.16 ± 0.03 a** | **0.11 ± 0.06 a** | **0.02 ± 0.01 b** |

| | | | | | |
|---|---|---|---|---|---|
| **Genus** | ***Halomonas*** | **0.21 ± 0.08 b** | **0.15 ± 0.06 b** | **0.23 ± 0.06 b** | **0.98 ± 0.37 a** |
| Genus | *Hyphomicrobium* | 0.06 ± 0.04 | 0.07 ± 0.04 | 0.05 ± 0.04 | 0.08 ± 0.04 |
| Genus | *Iamia* | 0.08 ± 0.06 | 0.21 ± 0.06 | 0.16 ± 0.06 | 0.23 ± 0.06 |
| Genus | *Ilumatobacter* | 0.37 ± 0.07 | 0.22 ± 0.08 | 0.30 ± 0.07 | 0.45 ± 0.07 |
| **Genus** | ***Jannaschia*** | **0.12 ± 0.04 a** | **0.02 ± 0.04 b** | **0.11 ± 0.04 a** | **0.09 ± 0.04 a** |
| **Genus** | ***Kocuria*** | **0.29 ± 0.10 b** | **0.36 ± 0.10 b** | **0.48 ± 0.10 a** | **0.76 ± 0.10 a** |
| Genus | *Kribbella* | 0.11 ± 0.05 | 0.12 ± 0.02 | 0.15 ± 0.08 | 0.17 ± 0.03 |
| Genus | *Legionella* | 0.02 ± 0.02 | 0.06 ± 0.02 | 0.06 ± 0.02 | 0.03 ± 0.02 |
| Genus | *Lewinella* | 0.11 ± 0.06 | 0.08 ± 0.06 | 0.06 ± 0.06 | 0.12 ± 0.06 |
| **Genus** | ***Litoreibacter*** | **0.08 ± 0.03 a** | **0.07 ± 0.05 a** | **0.00 ± 0.00 b** | **0.08 ± 0.07 a** |
| **Genus** | ***Lysobacter*** | **0.10 ± 0.08 b** | **0.38 ± 0.08 a** | **0.34 ± 0.08 a** | **0.27 ± 0.08 a** |
| Genus | *Marinobacter* | 0.20 ± 0.09 | 0.24 ± 0.09 | 0.16 ± 0.09 | 0.37 ± 0.09 |
| Genus | *Marinococcus* | 0.02 ± 0.06 | 0.01 ± 0.06 | 0.02 ± 0.06 | 0.2 ± 0.06 |
| Genus | *Marmoricola* | 0.20 ± 0.04 | 0.23 ± 0.04 | 0.18 ± 0.04 | 0.22 ± 0.04 |
| Genus | *Massilia* | 0.04 ± 0.05 | 0.04 ± 0.05 | 0.05 ± 0.05 | 0.1 ± 0.05 |
| **Genus** | ***Mesorhizobium*** | **0.07 ± 0.03 a** | **0.08 ± 0.03 a** | **0.05 ± 0.02 a** | **0.01 ± 0.00 b** |
| Genus | *Methylophaga* | 0.23 ± 0.06 | 0.13 ± 0.06 | 0.08 ± 0.06 | 0.22 ± 0.06 |
| **Genus** | ***Microlunatus*** | **0.00 ± 0.03 b** | **0.13 ± 0.03 a** | **0.02 ± 0.03 b** | **0.04 ± 0.03 b** |
| **Genus** | ***Microvirga*** | **0.45 ± 0.20 b** | **1.12 ± 0.20 a** | **0.71 ± 0.20 b** | **0.29 ± 0.20 b** |
| Genus | *Mycobacterium* | 0.52 ± 0.14 | 0.98 ± 0.14 | 0.63 ± 0.14 | 0.52 ± 0.14 |
| Genus | *Nitriliruptor* | 0.12 ± 0.07 | 0 ± 0.07 | 0.06 ± 0.07 | 0.25 ± 0.07 |
| Genus | *Nitrospira-genus* | 0.21 ± 0.06 | 0.34 ± 0.07 | 0.25 ± 0.07 | 0.21 ± 0.07 |
| Genus | *Nocardioides* | 0.42 ± 0.15 | 0.35 ± 0.05 | 0.41 ± 0.07 | 0.43 ± 0.09 |
| Genus | *Oceanicola* | 0.06 ± 0.04 | 0.034 ± 0.04 | 0.02 ± 0.03 | 0.14 ± 0.04 |
| Genus | *Ohtaekwangia* | 0.02 ± 0.03 | 0.08 ± 0.03 | 0.05 ± 0.03 | 0.02 ± 0.03 |
| Genus | *Ornithinimicrobium* | 0.17 ± 0.04 | 0.11 ± 0.04 | 0.13 ± 0.04 | 0.21 ± 0.04 |
| **Genus** | ***Paenibacillus*** | **0.06 ± 0.02 b** | **0.04 ± 0.02 b** | **0.04 ± 0.02 b** | **0.11 ± 0.02 a** |
| Genus | *Palleronia* | 0.19 ± 0.11 | 0.26 ± 0.11 | 0.53 ± 0.11 | 0.38 ± 0.11 |
| Genus | *Paracoccus* | 0.14 ± 0.09 | 0.47 ± 0.09 | 0.23 ± 0.09 | 0.29 ± 0.09 |
| Genus | *Patulibacter* | 0.11 ± 0.05 | 0.1 ± 0.05 | 0.16 ± 0.05 | 0.09 ± 0.05 |
| Genus | *Planococcus* | 0.056 ± 0.04 | 0.08 ± 0.04 | 0.17 ± 0.04 | 0.13 ± 0.04 |
| Genus | *Planomicrobium* | 0.08 ± 0.06 | 0.13 ± 0.06 | 0.15 ± 0.06 | 0.17 ± 0.06 |
| Genus | *Pontibacter* | 0.09 ± 0.05 | 0.12 ± 0.05 | 0.18 ± 0.05 | 0.06 ± 0.05 |
| Genus | *Porphyrobacter* | 0.01 ± 0.04 | 0.03 ± 0.04 | 0.06 ± 0.04 | 0.09 ± 0.04 |
| Genus | *Propionibacterium* | 0.03 ± 0.03 | 0.03 ± 0.03 | 0.1 ± 0.03 | 0.02 ± 0.03 |
| Genus | *Pseudomonas-genus* | 0.27 ± 0.07 | 0.21 ± 0.07 | 0.29 ± 0.07 | 0.30 ± 0.07 |
| **Genus** | ***Pseudonocardia*** | **0.50 ± 0.23 b** | **1.21 ± 0.23 a** | **1.20 ± 0.23 a** | **0.57 ± 0.23 b** |
| Genus | *Rheinheimera* | 0.28 ± 0.14 | 0.28 ± 0.14 | 0.69 ± 0.14 | 0.27 ± 0.14 |
| **Genus** | ***Rhizobium*** | **0.02 ± 0.03 b** | **0.25 ± 0.03 a** | **0.10 ± 0.03 b** | **0.09 ± 0.03 b** |
| **Genus** | ***Rhodoplanes*** | **0.03 ± 0.03 b** | **0.11 ± 0.03 b** | **0.20 ± 0.03 a** | **0.08 ± 0.03 b** |
| Genus | *Roseivivax* | 0.05 ± 0.03 | 0.04 ± 0.03 | 0.06 ± 0.03 | 0.07 ± 0.03 |
| Genus | *Rubellimicrobium* | 0.40 ± 0.08 | 0.30 ± 0.06 | 0.41 ± 0.23 | 0.32 ± 0.11 |
| **Genus** | ***Rubrobacter*** | **2.34 ± 0.21 a** | **2.26 ± 0.21 a** | **2.36 ± 0.21 a** | **1.81 ± 0.21 b** |

| | | | | | |
|---|---|---|---|---|---|
| **Genus** | ***Salegentibacter*** | **0.11 ± 0.12 b** | **0.34 ± 0.12 b** | **0.12 ± 0.12 b** | **0.68 ± 0.12 a** |
| **Genus** | ***Salinimicrobium*** | **1.26 ± 0.36 a** | **0.14 ± 0.36 b** | **1.68 ± 0.36 a** | **1.34 ± 0.36 a** |
| **Genus** | ***Skermanella*** | **0.12 ± 0.04 b** | **0.39 ± 0.09 a** | **0.41 ± 0.14 a** | **0.15 ± 0.04 b** |
| Genus | *Solirubrobacter* | 0.65 ± 0.13 | 0.82 ± 0.13 | 0.58 ± 0.13 | 0.46 ± 0.13 |
| Genus | *Sorangium* | 0.05 ± 0.03 | 0.07 ± 0.03 | 0.03 ± 0.03 | 0.01 ± 0.03 |
| Genus | *Steroidobacter* | 0.23 ± 0.05 | 0.16 ± 0.05 | 0.16 ± 0.05 | 0.07 ± 0.05 |
| Genus | *Streptomyces* | 0.25 ± 0.09 | 0.43 ± 0.09 | 0.20 ± 0.09 | 0.32 ± 0.09 |
| Genus | *Thermoleophilum* | 0.02 ± 0.03 | 0.07 ± 0.03 | 0.06 ± 0.03 | 0.1 ± 0.03 |
| Genus | *TM7-genus* | 0.50 ± 0.18 | 0.35 ± 0.18 | 0.84 ± 0.18 | 0.87 ± 0.18 |
| Genus | *Truepera* | 0.04 ± 0.05 | 0.08 ± 0.05 | 0.14 ± 0.05 | 0.06 ± 0.05 |
| **Genus** | ***Tumebacillus*** | **0.04 ± 0.03 b** | **0.17 ± 0.03 a** | **0.07 ± 0.03 b** | **0.08 ± 0.03 b** |
